# Frontloading HIV financing maximizes the achievable impact of HIV prevention

**DOI:** 10.1002/jia2.25087

**Published:** 2018-03-02

**Authors:** Sarah‐Jane Anderson, Peter D Ghys, Regina Ombam, Timothy B Hallett

**Affiliations:** ^1^ Department of Infectious Disease Epidemiology Imperial College London London UK; ^2^ UNAIDS Geneva Switzerland; ^3^ National AIDS Control Council Nairobi Kenya

**Keywords:** HIV Prevention, healthcare financing, health policy, mathematical modelling, programme planning

## Abstract

**Introduction:**

Due to the nature of funding, national planners and international donors typically balance budgets over short time periods when designing HIV programmes (˜5‐year funding cycles). We aim to explicitly quantify the cost of short‐term funding arrangements on the success of future HIV prevention programmes.

**Methods:**

Using mathematical models of HIV transmission in Kenya, we compare the impact of optimized combination prevention strategies under different constraints on investment over time. Each scenario has the same total budget for the 30‐year intervention period but the pattern of spending over time is allowed to vary. We look at the impact of programmes with decreasing, increasing or constant spending across 5‐year funding cycles for a 30‐year period. Interventions are optimized within each funding cycle such that strategies take a short‐term view of the epidemic. We compare these with two strategies with no spending pattern constraints: one with static intervention choices and another flexible strategy with interventions changed in year ten.

**Results and Discussion:**

For the same total 30‐year budget, greatest impact is achieved if larger initial prevention spending is offset by later treatment savings which leads to accumulating benefits in reduced infections. The impact under funding cycle constraints is determined by the extent to which greater initial spending is permitted. Short‐term funding constraints and funds held back to later years may reduce impact by up to 18% relative to the flexible long‐term strategy.

**Conclusions:**

Ensuring that funding arrangements are in place to support long‐term prevention strategies will make spending most impactful. Greater prevention spending now will bring considerable returns through reductions in new infections, greater population health and reductions in the burden on health services in the future.

## 1 Introduction

Renewed prevention efforts will be critical to future success in the control of the HIV epidemic. Despite substantial progress since the peak of the epidemic in 1997 1, HIV is still the leading cause of life years lost in Southern and Eastern Africa 2. Success in reducing the number of new infections has not been achieved everywhere, and many countries have seen an expansion or stabilization of the epidemic 3. But the increasing number of intervention tools and levels of commitment provide optimism for the future control of the HIV epidemic. This has culminated in the ambitious goal set by UNAIDS (Joint United Nations Programme on HIV/AIDS) of ending the epidemic, with a target of a 90% reduction in incidence from 2010 levels by 2030 4. Achieving such a target will depend heavily upon an intensification of prevention efforts and more strategic programming.

But the ability to allocate interventions efficiently is, in reality, constrained by a number of factors. The economic and political landscapes in which programmes are designed impose considerable limits on the flexibility of spending on HIV and the programmatic choices over time. National strategic plans and donor budgets focus on relatively short time periods, typically 5‐year cycles. Such a limited time frame makes designing strategies to meet long‐term targets considerably more challenging and may inhibit the most effective use of resources. Often, the amount of funds which a country will receive from national income and donor agencies is highly uncertain, further restricting the ability of national governments to look far into the future. However, the HIV epidemic is a long‐wave event 5, and the long‐term consequences of current programmatic choices must be assessed. The recent stabilization of the resources available for HIV programmes presents an important opportunity to revaluate these implications and plan future strategies 3.

Here, we aim to explicitly quantify the cost of short‐term funding cycles on the success of HIV prevention programmes. We focus on Kenya, a data‐rich country with a well characterized epidemic, for which we have developed mathematical models reflecting the epidemic and response across the country. Through using these models to project the impact of combination prevention strategies with different constraints on the pattern of investment over time, we can understand more about how funding arrangements influence the impact achievable.

## 2 Methods

### 2.1 Overview of Approach

Ethical approval was not required for this study. Here, we use mathematical modelling to explore how different constraints on the pattern of spending over time, and the time period over which outcomes are assessed, influence the impact of combination prevention programmes.

### 2.2 Outline of models

We use location specific mathematical models, which describe HIV transmission in each of the counties and major cities of Kenya individually (48 location specific models), to project the cost and impact of the future prevention interventions. Briefly, these models are compartmental deterministic dynamic transmission models which represent heterosexual and homosexual HIV transmission in a risk stratified population. These are described fully elsewhere 6.

### 2.3 Components of the combination prevention programme

For each of the funding strategies we compare, we identify the combination prevention strategy which will maximize infections averted under the budget constraints and time perspective particular to each scenario. All of the combination prevention programmes we describe can be composed of a number of different future candidate intervention components (Table 1), which can be applied differentially across populations and locations. We optimize the choice of interventions through projecting the cost and impact of all possible combinations of interventions, to find the strategy that maximizes infections averted within the budget constraints for each funding strategy.

**Table 1:** Characteristics of each component prevention intervention

Intervention	Coverage assumption	Efficacy assumption	Unit cost value used in the analysis^a^
Male circumcision	Scaled up at a fixed rate: intervention unable to exceed 80% of eligible men, based on VMMC targets set 18.	Risk of infection for circumcised man 60% less than for other men 19, 20.	$60 per circumcision 21
Behaviour change communication	The intervention is designed to reach 100% of the population‐with the adjustment in the partner change rate applied to the mean partner change rate in the entirety of the risk group.	20% reduction in risk for each low‐risk person reached, 50% reduction in risk for high risk groups (MSM and FSW).	$20 annually in FSW and MSM, $10 annually in the low risk population 21
Accelerated access to ART (Antiretroviral Therapy) i.e. active outreach for those with CD4 cell count > 350 cells per microlitre	Achievable coverage assumed to be 33% in low risk women and heterosexual men, 66% in FSW and MSM.	85% reduction in risk of transmission for a person on ART relative to others 22.	$515 annually 23, 24, 25
Pre‐exposure prophylaxis	The maximum PrEP coverage is assumed to be 25% in low risk women and heterosexual men and 50% in FSW and MSM.	75% reduction in risk of infection for a person on PrEP relative to others 24.	$250 annually 24

a Total costs of the programme are calculated using accounting identities.

### 2.4 Characteristics of the different funding strategies

All of the funding strategies we compare have the same total budget for HIV prevention and treatment ($39Bn) over the 30‐year period of the intervention (2015 to 2045). This total budget is broadly in line with an extrapolation of current projected future costs of the HIV programme in Kenya 7. This budget includes a projected $36Bn to cover treatment need (CD4 <350) in the absence of an intensified prevention programme, and a further $3Bn for the combination prevention programme under consideration.

While all strategies have the same total budget, we look at the effect of preallocating the funds differentially over time. The pattern of preallocation of funds across the different scenarios is given in Table 2. The $3Bn available for prevention spending is divided differentially across the years of the intervention period (as specified by the funding scenario). In all funding scenarios, the funds needed to cover treatment costs ($36Bn) are allocated over time based on the projected annual costs of treatment if there was no expanded combination prevention programme. However, when the different combination prevention programmes are applied in the model, the actual division of funds between prevention and treatment activities may differ from that preallocated. The greater the success of a prevention programme in averting infections, the lower the future treatment cost. Funds which are not spent on treatment are available for spending on prevention interventions instead, such that all strategies still spend the total budget ($39Bn). The degree to which each of the funding strategies are able to leverage these savings in treatment costs to fund prevention activities is dependent on the constraints on the pattern of prevention spending and the perspective of the optimization (whether a strategy considers long or short term outcomes).

**Table 2:** Characteristics of the different funding scenarios

Funding scenario	Total budget	Annual prevention spending	Flexibility in intervention choices	Perspective of optimization	Years 1 to 5	Years 6 to 10	Years 11 to 15	Years 16 to 20	Years 21 to 25	Years 26 to 30
Complete spending flexibility with change in interventions at year 10	$39Bn over the 30‐year period	Not Predefined: dependent on which intervention strategy is found to be optimal	Intervention choices can change once in the 30‐year period (at year 10)	Intervention choices optimized to those which avert the maximum number of infections for the whole 30‐year period	Prevention spending: 3Bn over the entire intervention period (not preallocated by 5‐year cycles) Treatment spending: $36Bn over the entire intervention period (not preallocated by 5‐year cycles)					
Complete spending flexibility	$39Bn over the 30‐year period	Not Predefined: dependent on which intervention strategy is found to be optimal	Intervention choices cannot change over the 30‐year period	Intervention choices optimized for the whole 30‐year period	Prevention spending: 3Bn over the entire intervention period (not preallocated by 5‐year cycles) Treatment spending: $36Bn over the entire intervention period (not preallocated by 5‐year cycles)					
Front‐Loaded Funding Cycles	$39Bn over the 30‐year period	Predefined: Decreasing prevention spending across the six 5‐year cycles	Intervention choices can change in each 5‐year cycle	Intervention choices optimized individually for each 5‐ year cycle	Prevention Spending: $857M Treatment Spending: $5340M	Prevention Spending: $714M Treatment Spending: $6181M	Prevention Spending: $571M Treatment Spending: $6385M	Prevention Spending: $428M Treatment Spending: $6290M	Prevention Spending: $286M Treatment Spending: $6119M	Prevention Spending: $143M Treatment Spending: $6001M
Equal Funding Cycles	$39Bn over the 30‐year period	Predefined: Equal prevention spending across the six 5‐year cycles	Intervention choices can change in each 5‐year cycle	Intervention choices optimized individually for each 5‐ year cycle	Prevention spending: $500M Treatment Spending: $5340M	Prevention spending: $500M treatment spending: $6181M	Prevention spending: $500M treatment spending: $6385M	Prevention spending: $500M treatment spending: $6290M	Prevention spending: $500M treatment spending: $6119M	Prevention spending: $500M treatment spending: $6001M
Back‐Loaded Funding Cycles	$39Bn over the 30‐year period	Predefined: increasing prevention spending across the six 5‐year cycles	Intervention choices can change in each 5‐year cycle	Intervention choices optimized individually for each 5‐ year cycle	Prevention Spending: $143M Treatment Spending: $5340M	Prevention Spending: $286M Treatment Spending: $6181M	Prevention Spending: $428MTreatment Spending: $6385M	Prevention Spending: $571M Treatment Spending: $6290M	Prevention Spending: $714M Treatment Spending: $6119M	Prevention Spending: $857M Treatment Spending: $6001M

The long‐term “flexible” strategies (Table 2, row 1 and 2) seek to maximize the infections averted over the entire 30‐year period, with no constraints on the pattern of annual expenditure over time. In one strategy, interventions must be maintained for the entire 30‐year period (Table 2, row 2), in another where they can change at year ten (Table 2, row 1). Both of these approaches assess costs and outcomes over the entire 30‐year intervention period when optimizing intervention choices, and as a result are able to offset earlier prevention spending with savings in treatment funds later in the 30‐year intervention period.

Three further strategies explore the effect of funding cycles and their implied short‐term intervention choices on the achievable impact and distribution of spending over the course of the intervention (Table 2, row 3 to 5). The $3Bn prevention expenditure for the 30‐year intervention period is preallocated between constituent 5‐year blocks, with scenarios considering (1) back‐loaded funding (2) equal funding and (3) front‐loaded funding over time. In every funding cycle, the total spend includes the projected treatment need for that block and the allocated prevention spending. For each 5‐year block of the intervention period, the choice of prevention interventions is optimized to maximize infections averted that 5‐year block. Those intervention choices are fixed and the optimal interventions for the next 5‐year block assessed. In this way the strategy considers short‐term strategies chosen based on what is optimal for the 5‐year block of interest only, and does not look ahead to the end of the full 30‐year period. As a result, these strategies have a much lower ability to leverage savings in future treatment costs to invest in prevention.

## 3 Results and Discussion

**Figure 1:**
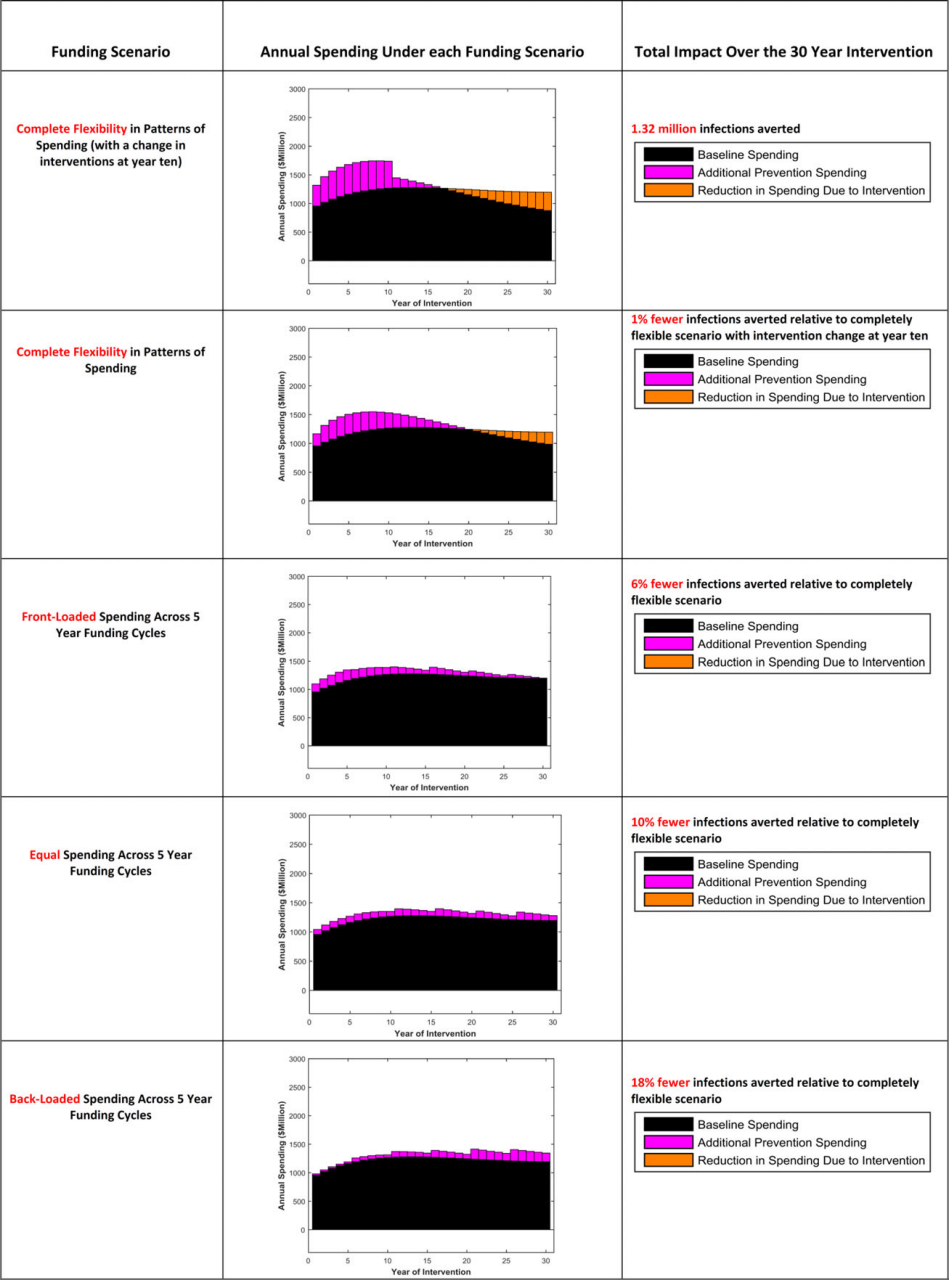
The profile of annual spending and total impact achievable under each funding scenario. Baseline spending refers to the projected cost of treatment to all at late stages of disease if the programme is maintained and prevention efforts are not intensified.

Figure 1 presents the pattern of spending over time and resulting number of infections averted under each funding scenario.

The strategy which was found to avert the greatest number of infections was the completely flexible scenario with a change in intervention choices at year ten (Figure 1, row 1). This approach considers the long‐term (30 years) outcomes of the prevention programme. Through allowing substantial prevention spending in early years to be offset by later savings in treatment costs (highlighted as reduction in treatment spending due to intervention), 1.32 million infections can be averted over the 30‐year period. As this approach allows for intervention choices to change at year ten, a greater number of interventions are employed and there are higher associated annual costs in this first 10‐year period than under the flexible approach without intervention change. However, this leads to only marginal improvement in impact over the flexible scenario with no intervention change (Figure 1, row 2), suggesting that a change in intervention strategies offers only limited additional impact.

We observe that all of the funding cycle approaches (Figure 1, row 3 to 5) generate less impact than the flexible approaches as they are forced to respect spending constraints over short‐term periods, and are less able to leverage future savings in treatment costs to allow for greater prevention spending now. The impact achievable by these strategies is dependent on the size of the immediate investment in prevention which is possible. Across the funding cycle strategies the more restricted the frontloading of expenditure, the lower the impact achievable, with 18% fewer infections averted under the back‐loaded funding cycle scenario (where spending increases over the cycles) relative to the completely flexible scenario with interventions changing in year ten.

**Figure 2:**
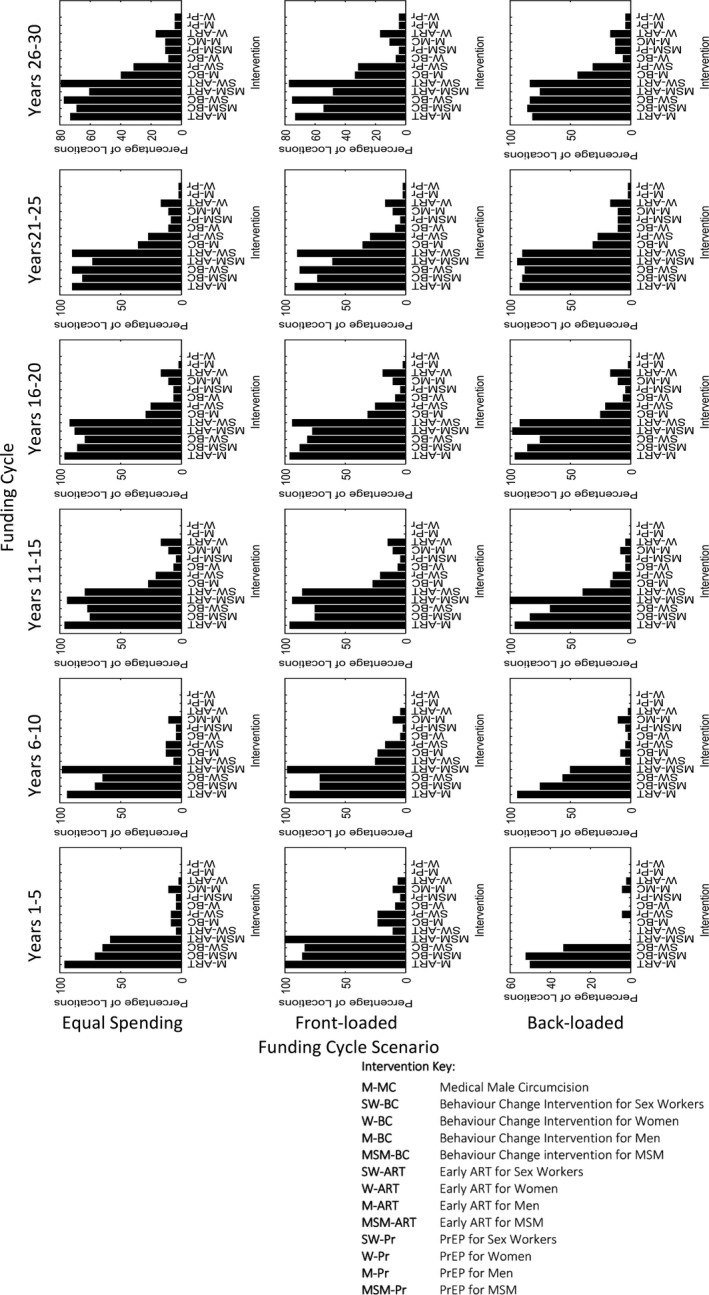
The percentage of locations implementing each intervention by risk population, across each of the six funding cycles (columns) under the three different funding cycle scenarios (rows).

We also explore how the choice of interventions changes across funding cycles (Figure 2), through examining the percentage of locations implementing each intervention by risk population across each of the six funding cycles under the three different funding cycle approaches. Across all cycles, we observe that a number of interventions are consistently more favourable for implementation than others (e.g. Behaviour Change in FSW and MSM, accelerated access to ART in men and MSM), suggesting the order of priority for rollout of the different prevention interventions is conserved over time. Instead, the difference in impact between the funding cycle approaches is due to intensity of the programme in each funding cycle (how many interventions can be applied) given the preallocated funds available.

While Figure 1 gives the cumulative infections averted over the entire intervention period, we can also look at differences in the annual number of infections at different years. The greatest difference in annual infections between the funding scenarios occurs early in the interventions period, with the completely flexible scenario with intervention change achieving 82% fewer annual new infections in 2020 relative to 2010 levels, compared to the back‐loaded funding cycle approach achieving 58% fewer. The UNAIDS Fast‐Track targets include the reduction in annual new infections by 90% in 2030 from 2010 levels. None of the funding strategies we include achieve this, with the back‐loaded, equal and front‐loaded funding cycle strategies achieving 78%, 83% and 84% reductions respectively, and the flexible approaches, with and without intervention change, achieving 87% and 88% reductions. By 2040, all strategies have reduced annual new infections relative to 2010 by greater than 89% (89 to 92% across strategies).

The call from UNAIDS to “Fast‐Track” the response, with rapid immediate scale up of prevention efforts and treatment provision, heralds an important push to end the HIV epidemic 4, 8. The analysis presented here provides further evidence that such frontloading of investment will be essential to generating greatest impact. Indeed, other modelling studies suggests that immediate rapid expansion of efforts, particularly until 2020, will be critical to meeting the goal of ending the epidemic by 2030 4, 9. At the national level, modelling studies have also found that intensifying investment in prevention and treatment immediately will be most impactful 10. Intensifying the response is critical as the large and growing treatment costs are, in some places, already at the same scale as national debt, with needs exceeding the projected HIV fiscal space in the long‐term 11.

Here, we demonstrate how current funding arrangements (short‐term funding cycles) restrict the impact of prevention programmes through not allowing programmes to leverage future treatment savings for greater investment in prevention immediately. This is particularly important in situations where funding is “held back” until a later date.

Examining how the components of the intervention package would optimally change over time periods, highlights how impact is attributable to this increased investment earlier in the epidemic rather than changes in the relative cost‐effectiveness of different intervention. In this way, the results suggest that having the flexibility to change interventions over time is not as important as intensifying intervention efforts early on and maximizing the front‐loading of investment.

This tension between the need for long‐term planning and short‐term funding cycles is thought to have shaped the response, and led to the relatively limited use of strategies with benefits apparent over the longer term (e.g. structural interventions) 12. Actually facilitating a frontloading pattern of investment however will present a significant financial challenge. There is often considerable uncertainty around the availability of funds and budget allocation in the long‐term, restricting the ability of policymakers to plan into the future. Short‐term funding cycles are the norm, yet the funding cycle approach presented here demonstrates how such restrictions on the pattern of spending over time can substantially reduce the impact of HIV prevention strategies. To allow for greater upfront investment all new funding sources should be explored, including raising domestic commitments, innovative financing strategies or leveraging cross‐sectorial benefits as has been suggested for structural interventions 13, 14, 15. However, many countries face a plethora of challenges across the health sector and beyond and will have a large number of different priorities and sources of funding, restricting their ability to immediately scale up programmes.

A number of extensions to this analysis could be considered. Future analyses could look within funding cycles, modifying the annual pattern of disbursement to provide direct guidance to policy makers under these constraints. The application of discount rates may lessen the priority on immediate investment; however this analysis finds it is early spending which has most impact on the epidemic. These analyses could also be modified to account for the potential introduction of new interventions currently under development. Greater frontloading of funding may restrict funds available for investment in future technologies. However, previous analysis have suggested that scaling up existing interventions is still critical and new interventions address remaining gaps 16. Although assumed constant here, the unit costs of interventions may vary over time, and are likely to depend on the scale of the programme. Costs will also be dependent on other factors such as synergies between interventions, the setting of the service and channels of delivery. Future costing studies are needed to generate more specific cost estimates. Furthermore, the wider returns for national investment in prevention could be quantified; including productivity gains and the averted costs of orphan care, and will be critical to presenting the value of intervention efforts 17. The extent to which prevention spending is offset by benefits will be greater the longer into the future outcomes are considered.

## 4 Conclusions

This study highlights the need to take a long‐term view when designing HIV programmes. Intensifying current prevention efforts will be offset by later savings in treatment costs and will lead to greater heath and fewer new infections in the population. Short‐term funding cycles inhibit optimal allocation and reduce impact considerably and this must be recognized when planning, designing and implementing future prevention programmes.

## Competing interests

SJA reports personal fees from The Bill and Melinda Gates Foundation, personal fees from Avenir Health, personal fees from Anansi Health, and personal fees from the Global Fund outside the submitted work. TBH received grants and personal fees from the Bill & Melinda Gates Foundation during the conduct of the study; grants and personal fees from World Bank; grants from UNAIDS, and The Rush Foundation; personal fees from the University of Washington, New York University, and Global Fund outside of the submitted work. For the remaining authors, none were declared.

## Authors' contributions

SJA and TBH conceived the study and developed the methods and analysis. SJA wrote the first draft of the paper. PDG and RO advised on the interpretation of the analysis. All authors contributed to the writing of the manuscript and reviewed and approved the final version.
